# Neoadjuvant chemotherapy with different dose regimens of docetaxel, cisplatin and fluorouracil (TPF) for locoregionally advanced nasopharyngeal carcinoma: a retrospective study

**DOI:** 10.18632/oncotarget.21992

**Published:** 2017-10-20

**Authors:** Ting Jin, Qun Zhang, Feng Jiang, Wei-Feng Qin, Qi-Feng Jin, Cai-Neng Cao, Yong-Feng Piao, Xing-Lai Feng, Wei Luo, Xiao-Zhong Chen

**Affiliations:** ^1^ Key Laboratory of Radiation Oncology in Zhejiang Province, Hangzhou, Zhejiang 310022, People’s Republic of China; ^2^ Department of Radiation Oncology, Zhejiang Cancer Hospital, Hangzhou, Zhejiang 310022, People’s Republic of China; ^3^ Department of Radiation Oncology, The First Affiliated Hospital, Sun Yat-Sen University, Guangzhou, Guangdong 510060, People’s Republic of China; ^4^ Department of Radiation Oncology, Sun Yat-Sen University Cancer Centre, State Key Laboratory of Oncology in South China, Collaborative Innovation Centre for Cancer Medicine, Guangzhou, Guangdong 510060, People’s Republic of China

**Keywords:** nasopharyngeal carcinoma, neoadjuvant chemotherapy, concurrent chemoradiation, cisplatin, docetaxel

## Abstract

**Objective:**

Compare high- vs. low-dose TPF neoadjuvant chemotherapy with chemoradiotherapy in Chinese patients with locoregionally advanced nasopharyngeal carcinoma (NPC).

**Materials and Methods:**

Retrospective analysis of 210 stage III/IV NPC patients treated between April 1, 2012 and April 1, 2014; 138 received three cycles of high-dose TPF (H-TPF) every 3 weeks at Zhejiang Cancer Hospital and 72, three cycles of low-dose TPF (L-TPF) every 3 weeks at Sun Yat-Sen University Cancer Center. H-TPF was docetaxel (75 mg/m^2^; 1 h infusion), cisplatin (75 mg/m^2^; 0.5–3 h), then 5-fluorouracil (600 mg/m2/day; 4 days). L-TPF was docetaxel (60 mg/m^2^), cisplatin (65 mg/m^2^), then 5-fluorouracil (550 mg/m^2^/day; 5 days). All patients received chemoradiotherapy.

**Results:**

During neoadjuvant chemotherapy, treatment delays were more frequent for H-TPF than L-TPF (33.3% vs. 19.4%; *P* = 0.034). During chemoradiotherapy, grade III–IV anemia, thrombocytopenia and neutropenia were more common for H-TPF than L-TPF (*P* < 0.001, *P* < 0.001, *P* = 0.048). Fewer patients in the H-TPF group finished two cycles of concurrent chemotherapy (81.2% vs. 100%, *P* < 0.001). Three-year PFS (84.5% vs. 80.6%, *P* = 0.484) and OS (91.1% vs. 93.5%, *P* = 0.542) were not significantly different between H-TPF and L-TPF.

**Conclusions:**

L-TPF neoadjuvant chemotherapy has substantially better tolerance and compliance rates and similar treatment efficacy to H-TPF neoadjuvant chemotherapy in locoregionally-advanced NPC.

## INTRODUCTION

Approximately 86,700 new cases of nasopharyngeal carcinoma (NPC) were diagnosed worldwide in 2012, and the disease has an extremely unbalanced, endemic distribution. The highest incidences are reported in southeast Asia, Micronesia and Polynesia, eastern Asia, and northern Africa [1]. According to the 6th edition of the American Joint Commission on Cancer (AJCC) staging system, 60–70% of patients present with stage III–IVB NPC [2, 3]. The combined use of magnetic resonance imaging (MRI), intensity-modulated radiotherapy (IMRT) and concurrent chemoradiotherapy (CCRT), has substantially improved treatment outcomes, especially locoregional control, in locoregionally advanced NPC; the 5-year locoregional control rate for stage III–IVB NPC ranges from 89.7–93.6% [4–6]. At present, distant metastasis is the main cause of treatment failure, with a 5-year distant *metastasis-*free survival (DMFS) rate of 82.6% for stage III–IVB NPC [4, 7].

One strategy to improve distant control in patients at high risk of distant failure is a neoadjuvant-concurrent sequence. Several factors support this therapeutic sequence: NPC is chemosensitive, with an objective response rate of 85–89% after neoadjuvant chemotherapy in stage III–IVB NPC [8, 9]; early use of a potent combination of cytotoxic drugs at their full doses would theoretically eradicate micrometastases more effectively and reducing tumor and cervical lymph node volumes may facilitate radiotherapy; and neoadjuvant chemotherapy is better tolerated than adjuvant chemotherapy [10–13]. In 2016, Ying Sun et al. [14] reported a multicenter, randomized controlled phase 3 trial that compared the efficacy of TPF neoadjuvant chemotherapy plus CCRT with CCRT alone in locoregionally advanced NPC. Compared to CCRT alone, TPF neoadjuvant chemotherapy followed by CCRT significantly increased failure-free survival, overall survival (OS), and distant failure-free survival. However, only 30% of patients in the neoadjuvant chemotherapy plus CCRT group completed three cycles of concurrent cisplatin during CCRT, mainly due to treatment toxicities and patient refusal.

As far as we are aware, the optimal neoadjuvant chemotherapy TPF regimen dose-intensity for patients with locoregionally advanced NPC remains unknown. Therefore, we carried out this retrospective study to compare the side effects and efficacy of a high dose regimen of TPF (H-TPF) + CCRT with a low dose regimen of TPF (L-TPF) + CCRT in Chinese patients with locoregionally advanced NPC.

## MATERIALS AND METHODS

### Patients

A total of 210 patients with NPC treated with neoadjuvant chemotherapy plus CCRT at Zhejiang Cancer Hospital or Sun Yat-Sen University Cancer Center between April 1, 2012 and April 1, 2014 were analyzed retrospectively. The inclusion criteria included pathologically confirmed non-metastatic, histologically proven non-keratinizing stage III or IV NPC without distant metastasis, in addition to a Karnofsky performance score ≥ 70; age 18–70 years; and adequate bone marrow function (hemoglobin ≥ 80 g/L; white blood cells ≥ 4.0 × 10^9^/L; absolute neutrophil count ≥ 2.0 × 10^9^/L, platelets ≥ 100 × 10^9^/L), renal function (creatinine clearance > 60 ml/min) and hepatic function (aspartate aminotransferase/alanine aminotransferase ≤ 1.5 × upper limits of normal). Patients who previously received radiotherapy or chemotherapy, or had other cancers, cardiac arrhythmia, coronary heart disease, peripheral neuropathy, or psychiatric disorders/psychological conditions that may adversely affect treatment compliance were excluded. Pregnant or lactating females and females of childbearing age who lacked effective contraception were also excluded. Written informed consent was obtained from the individual patients and the experimental protocol was approved by Zhejiang Cancer Hospital Institutional Review Board (Hangzhou, China) and Sun Yat-Sen University Cancer Center Review Board (Guangzhou, China).

### Radiotherapy

The target volumes and organs at risk for each individual patient were delineated according to International Commission on Radiation Units and Measurements Reports 50 and 62. All patients received one fraction of IMRT daily for 5 consecutive days per week. The prescribed radiation doses for the patients treated at Zhejiang Cancer Hospital were 63–70.4 Gy at 2.1–2.3 Gy/fraction over 30–32 fractions to the planning target volume (PTV) of the GTVnx (primary nasopharyngeal gross tumor volume) and GTVnd (involved cervical lymph nodes), with 60–60.8 Gy to the PTV of CTV1 (high-risk regions) and 54–54.4 Gy to the PTV of CTV2 (low-risk regions and neck nodal regions). The prescribed radiation doses for patients treated at Sun Yat-Sen University Cancer Center were 63–70 Gy at 2.1–2.12 Gy/fraction over 30–33 fractions to the planning target volume (PTV) of the GTVnx and GTVnd, with 60 Gy to the PTV of CTV1 (high-risk regions) and 54 Gy to the PTV of CTV2 (low-risk regions and neck nodal regions).

### Chemotherapy

Patients at Zhejiang Cancer Hospital were treated with three cycles of a high-dose regimen of TPF (H-TPF) neoadjuvant chemotherapy every 3 weeks. Patients at Sun Yat-Sen University Cancer Center were treated with three cycles of a low dose regimen of TPF (L-TPF) neoadjuvant chemotherapy every 3 weeks. H-TPF was docetaxel (75 mg/m^2^) administered as a 1 h intravenous infusion, followed by intravenous cisplatin (75 mg/m^2^) over 0.5 to 3 h, then 5-fluorouracil (600 mg/m^2^/day) as a continuous 24 h infusion for 4 days. L-TPF was docetaxel (60 mg/m^2^) administered as a 1 h intravenous infusion, followed by intravenous cisplatin (65 mg/m^2^) over 0.5 to 3 h, then 5-fluorouracil (550 mg/m^2^/day) as a continuous 24 h infusion for 5 days.

During radiotherapy, patients at both Zhejiang Cancer Hospital and Sun Yat-Sen University Cancer Center received cisplatin (80 mg/m^2^) every three weeks as concurrent chemotherapy. Dose modifications for the H-TPF group during NACT are prescribed in our previous study [15]. Dose modifications for docetaxel and cisplatin in the L-TPF group during NACT were same as for the H-TPF group. Dose modifications for fluorouracil in the L-TPF group during NACT were different to those of the H-TPF group. In the L-TPF group, fluorouracil was reduced by 110 mg/m^2^ for grade 3 diarrhea lasting for less than 3 days, and chemotherapy was stopped permanently if grade 4 toxic effects developed or grade 3 diarrhea lasted more than 3 days.

### Follow-up

Patients were followed-up every 3 months during the first 2 years, every 6 months for the next 3 years, and then annually thereafter until death. OS and PFS were recorded. The details of our follow-up protocol are described in our previous study [15].

### Statistical analysis

Statistical analyses were performed using SPSS 16.0 (SPSS, Chicago, IL, USA). The difference in the frequencies of individual category between groups were analyzed using the Chi-square test. Survival was estimated using the Kaplan-Meier method and analyzed with the log-rank test. All *P-*values are two-tailed and *P* < 0.05 was considered statistically significant.

## RESULTS

### Patients

Between April 1, 2012 and April 1, 2014, 138 eligible patients treated at Zhejiang Cancer Hospital and 72 patients treated at Sun Yat-Sen University Cancer Center in China were enrolled. The cutoff date for analysis was April 1, 2016 (2 years follow-up for the last patient enrolled; median, 36 months; range, 24–48 months). The groups receiving L-TPF and H-TPF were well-balanced in terms of baseline demographic and clinicopathological characteristics, except for TNM stage (Table 1). The H-TPF group contained a lower number of patients with stage IV NPC than the L-TPF group (37.7% vs. 63.9%, *P* < 0.001).

**Table 1:** Baseline characteristics of the 210 patients with locoregionally-advanced nasopharyngeal cancer in each treatment arm

Variable	H-TPF + CCRT (*n* = 138)	L-TPF + CCRT (*n* = 72)	*P*-value^*^
Sex			0.345
Male	99 (71.7)	56 (77.8)	
Female	39 (28.3)	16 (22.2)	
Age, years			
Median	48	44	
Range	18–68	18–68	
Karnofsky performance score			0.943
100–90	130 (94.2)	68 (94.4)	
80–70	8 (5.8)	4 (5.6)	
T category			0.604
T 1–2	25 (18.1)	11 (15.3)	
T 3–4	113 (81.9)	61 (84.7)	
N category			0.215
N 0–1	48 (34.8)	19 (26.4)	
N 2–3	90 (65.2)	53 (73.6)	
Stage			< 0.001
III	86 (62.3)	26 (36.1)	
IVA-B	52 (37.7)	46 (63.9)	

^*^Calculated using the χ^2^ test. Values are reported as *n* (%).

### Treatment and dose modifications

All 210 patients (100%) started neoadjuvant chemotherapy (Table 2). In the L-TPF group, docetaxel was decreased to 48 mg/m^2^ in the second cycle for five patients because of grade 4 neutropenia and/or thrombocytopenia, and fluorouracil was decreased by 110 mg/m^2^ for six patients due to grade 3 mucositis or diarrhea. In the H-TPF group, docetaxel was decreased to 60 mg/m^2^ in the second cycle for 60 patients because of grade 4 neutropenia and/or thrombocytopenia, and cisplatin was decreased to 60 mg/m^2^ in the third cycle for seven patients due to grade 4 neutropenia and/or thrombocytopenia after docetaxel, and fluorouracil decreased by 120 mg/m^2^ for 11 patients due to grade 3 mucositis or diarrhea. During CCRT, only 81.2% of patients in the H-TPF group completed two cycles of concurrent cisplatin whereas 100% of patients in the L-TPF group completed two cycles of concurrent cisplatin (*P* < 0.001).

**Table 2:** Dose modifications and treatment delays during induction chemotherapy

	H-TPF (*n* = 138)	L-TPF (*n* = 72)	*P*-value^*^
Dose modifications during induction chemotherapy			
Docetaxel	60 (43.5)	5(6.9)	< 0.001
Cisplatin	7 (5.1)	0 (0)	0.124
Fluorouracil	11 (8.0)	6 (8.4)	0.927
Treatment delays during induction chemotherapy^**^			
Patients who experienced delays, *n* (%)	46 (33.3)	14 (19.4)	0.034
Reason for delay			
Hematologic	26 (18.8)	8(11.1)	0.149
Non-hematologic	10 (7.2)	3 (4.2)	0.564
Other^***^	10 (7.2)	3 (4.2)	0.564

*Calculated using the χ^2^ test.

**We defined treatment delays as a delay or 2 or more days to the next cycle of neoadjuvant chemotherapy.

***Including personal reasons and vacations.

### Efficacy

**Figure 1:**
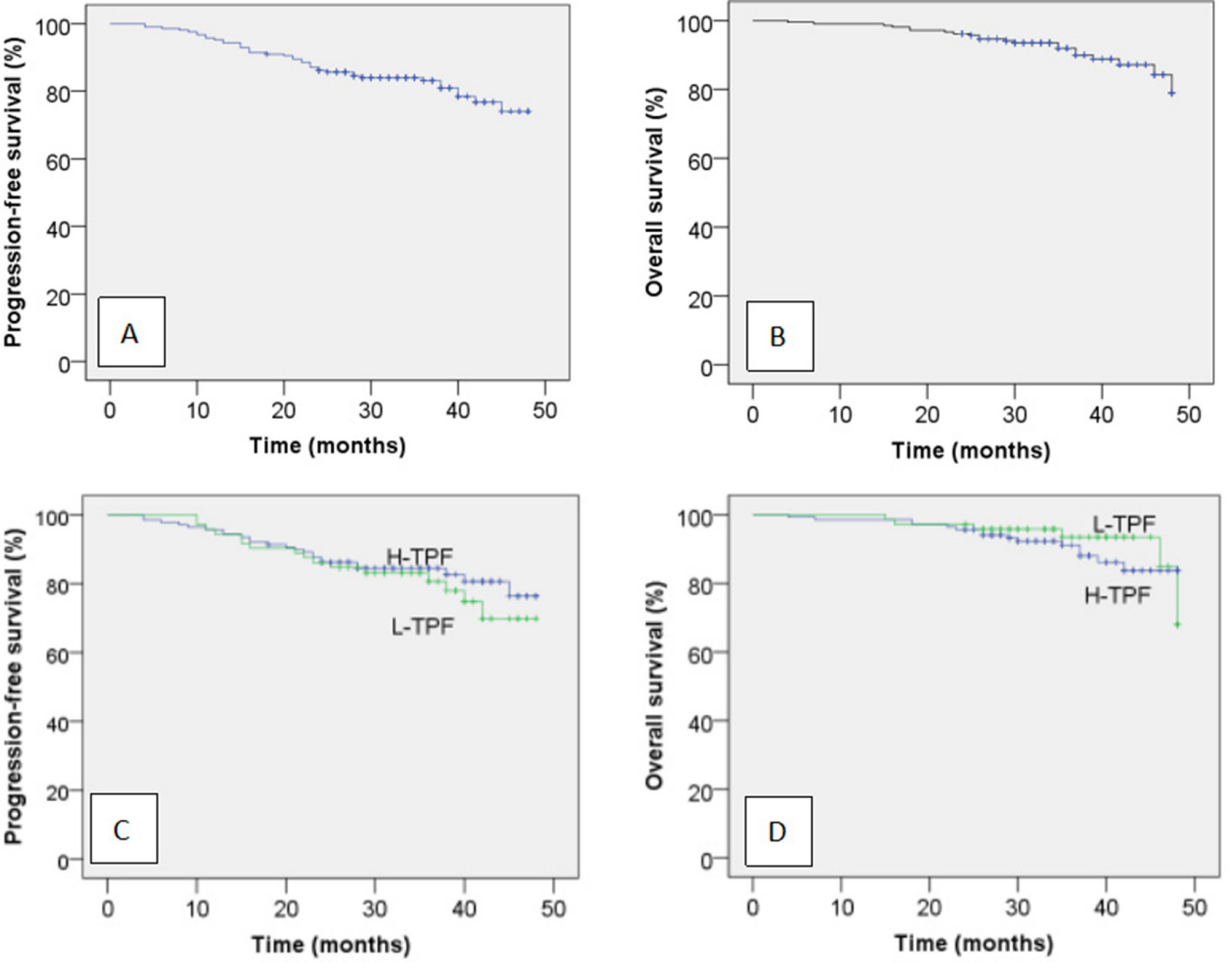
Kaplan-Meir progression-free survival (A) and overall survival curves (B) for all 210 patients with locoregionally advanced NPC and progression-free survival (C) and overall survival curves (D) for the patients stratified by neoadjuvant chemotherapy regimen

Overall, 3-year PFS and OS for the entire cohort were 83.1% and 91.9%, respectively (Figure 1A–1B). For the H-TPF group, median OS was 36 months; 2- and 3-year OS were 95.7% and 91.1%, respectively. For the L-TPF group, median OS was 36 months; 2- and 3-year OS were 97.2% and 93.5%. For the H-TPF group, median PFS was 34.5 months; 2- and 3-year PFS were 86.2% and 84.5%. For the L-TPF group, median PFS was 35 months; 2- and 3-year PFS were 86.1% and 80.6%. PFS or OS were not significantly different between the H-TPF and L-TPF groups (3-year PFS 84.5% vs. 80.6%, *P* = 0.484; 3-year OS 91.1% vs. 93.5%, *P* = 0.542; Figure 1C–1D). Two and 3-year distant metastasis free survival (DMFS) were 92.0% and 91.1% for the H-TPF group and 90.3% and 84.8% for the L-TPF group. A DMFS benefit was not observed for H-TPF compared to L-TPF (*P* = 0.170).

Overall, 21/210 patients (10%) died: 15/138 (10.7%) in the H-TPF group and 6/72 (8.3%) in the L-TPF group., Two- and 3-year DMFS were 92.0% and 91.1% for the H-TPF group and 90.3% and 84.8% for the L-TPF group, respectively. DMFS was not significantly different between the H-TPF and L-TPF groups (*P* = 0.170). Tumor progression was the cause of the death for all patients who died. Twenty patients in the H-TPF group and 16 patients in the L-TPF group suffered treatment failure. Ten patients in the H-TPF group developed local recurrence, four developed regional recurrence, and 12 developed distant metastases. Four patients in the L-TPF group developed local recurrence, four developed regional recurrence and 11 developed distant metastases (Table 3).

**Table 3:** Comparison of the treatment outcomes of the induction chemotherapy regimens

Variable	H-TPF + CCRT (*n* = 138)	L-TPF + CCRT (*n* = 72)	χ2	*P*-value^*^
Progression-free survival			0.490	0.484
Median duration, months	34.5	35		
Rate, %				
Two-year	86.2	86.1		
Three-year (estimated)	84.5	80.6		
Overall survival			0.372	0.542
Median duration, months	36	36		
Rate, %				
At 2 years	95.7	97.2		
At 3 years	91.1	93.5		
Sites of treatment failure				
Locoregional failure, *n* (%)				
Primary	10 (7.2)	4 (5.6)		
Neck	4 (2.9)	4 (5.6)		
Distant metastases, *n* (%)				
Distant	12 (8.7)	11 (15.3)		

^*^Calculated using the Kaplan–Meier method.

### Adverse events

The frequencies of grade 3 or 4 febrile neutropenia, anemia and thrombocytopenia were similar between groups. Grade 4 neutropenia occurred in 43.4% of patients in the H-TPF group and 6.9% of the L-TPF group (*P* < 0.001). Grade 1 or 2 liver dysfunction occurred in 50.7% of patients in the H-TPF group and 41.6% of the L-TPF group (*P* = 0.068; Table 4). There were no significant differences in non-hematologic adverse events between groups during neoadjuvant chemotherapy (Table 4). More patients in the H-TPF group experienced treatment delays compared to the L-TPF group (33.3% vs. 19.4%, *P* = 0.034; Table 2).

**Table 4:** Adverse events and treatment delays

	H-TPF + CCRT (*n* = 138)	L-TPF + CCRT (*n* = 72)	*P*-value^*^
Adverse events during induction chemotherapy, *n* (%)			
Hematologic			
Anemia (grade 3 or 4)	3 (2.2)	1 (1.4)	> 0.999
Thrombocytopenia (grade 3 or 4)	3 (2.2)	0 (0)	> 0.999
Neutropenia (grade 3 or 4)	88 (63.8)	53 (73.6)	0.149
Febrile neutropenia	14 (10.1)	5 (6.9)	0.443
Non-hematologic (grade 3 or 4)			
Stomatitis (mucositis)	3 (2.2)	4 (5.6)	0.373
Nausea	12 (8.7)	2 (2.8)	0.180
Vomiting	6 (4.3)	2 (2.8)	0.854
Diarrhea	10 (7.2)	6 (8.3)	0.778
Fatigue	15 (10.9)	4 5.6)	0.203
Anorexia	10 (7.2)	4 (5.6)	0.861
Liver dysfunction (grade 1 or 2)	70 (50.7)	27 (41.6)	0.068
Kidney dysfunction (grade 1 or 2)	3 (2.2)	6 (8.3)	0.083
Adverse events during chemoradiotherapy			
Hematologic			
Anemia (grade 3 or 4)	34 (24.6)	1 (1.4)	< 0.001
Thrombocytopenia (grade 3 or 4)	32 (23.2)	2 (2.8)	< 0.001
Neutropenia (grade 3 or 4)	49 (35.5)	16 (22.2)	0.048
Febrile neutropenia	5 (3.6)	2 (2.8)	> 0.999
Non-hematologic (grade 3 or 4)			
Stomatitis (mucositis)	30 (21.7)	18 (25.0)	0.593
Nausea	11 (8.0)	7 (9.7)	0.667
Vomiting	9 (6.5)	7 (9.7)	0.407
Diarrhea	2 (1.4)	1 (1.4)	> 0.999
Fatigue	20 (14.5)	7 (9.7)	0.327
Anorexia	28 (20.3)	10 (13.9)	0.253
Dermatitis	14 (10.1)	11 (15.3)	0.276
Esophagitis, dysphagia or odynophagia	5 (3.6)	11 (15.3)	0.003
Dry mouth	7 (5.1)	10 (13.9)	0.026
Liver dysfunction (grade 1 or 2)	62 (44.9)	10 (13.9)	< 0.001
Kidney dysfunction (grade 1 or 2)	44 (31.9)	1 (1.4)	< 0.001
Cycles of concurrent chemotherapy			< 0.001
One	26 (18.8)	0 (0)	
Two	112 (81.2)	72 (100)	

^*^Calculated using the χ^2^ test.

During chemoradiotherapy, grade 3 or 4 anemia occurred in 24.6% of patients in the H-TPF group and 1.4% of the L-TPF group (*P* < 0.001), grade 3 or 4 thrombocytopenia occurred in 23.2% of the H-TPF group and 2.8% of the L-TPF group (*P* < 0.001), and grade 3 or 4 neutropenia occurred in 35.5% of the H-TPF group and 22.2% of the L-TPF group (*P* = 0.048; Table 4). There were higher frequencies of grade 1 or 2 liver dysfunction and kidney dysfunction in the H-TPF group than the L-TPF group during chemoradiotherapy (*P* < 0.001). With the exception of significantly higher frequencies of esophagitis, dysphagia or odynophagia, and dry mouth in the L-TPF group, there were no major differences in non-hematologic adverse events between groups during chemoradiotherapy (Table 4).

## DISCUSSION

A recent multicenter, randomized controlled phase 3 trial among patients with locoregionally advanced NPC demonstrated NACT + CCRT significantly increased failure-free survival, OS and distant failure-free survival, but not locoregional failure-free survival, compared to CCRT alone [14]. However, NACT + CCRT significantly impaired locoregional control due to delays in CCRT caused by the higher toxicity of NACT [16]. Therefore, selection of the optimal TPF dose regimen is crucial. This study indicates H-TPF neoadjuvant chemotherapy followed by CCRT is not superior to L-TPF neoadjuvant chemotherapy followed by CCRT in terms of OS or PFS. Moreover, L-TPF neoadjuvant chemotherapy had substantially better tolerance and compliance rates than H-TPF neoadjuvant chemotherapy.

As there is no unified standard or consensus, doctors at different hospitals in China use varied dose regimens for TPF neoadjuvant chemotherapy. In 2013, Lin Kong et al. [17] reported 3-year PFS rates of 78.2% for 52 patients with stage III NPC and 85.1% for 64 patients with stage IVA/IVB NPC who received TPF (75 mg/m^2^ docetaxel, 75 mg/m^2^ cisplatin, 2500 mg/m^2^ 5-fluorouracil every 3 weeks for three cycles) followed by cisplatin 40 mg/m^2^ per week concurrently with 3D-CRT or IMRT. The PFS and OS rates for the H-TPF group in this study are similar to those reported by Lin Kong et al. [17]: 84.5% vs. 78.2–85.1% and 91.1% vs. 90.2–94.8%. In 2016, Ying Sun et al. [14] reported 3-year PFS and OS rates of 80.0% and 92.0% for 241 patients with T3–4N1/N2–3M0 NPC who received TPF (60 mg/m^2^ docetaxel, 60 mg/m^2^ cisplatin, 3000 mg/m^2^ 5-fluorouracil every 3 weeks for three cycles) followed by 100 mg/m^2^ cisplatin every 3 weeks concurrently with IMRT. The PFS and OS rates for the L-TPF group in this study are similar to those reported by Ying Sun et al. [14]: 80.6% vs. 80% and 93.5% vs. 92%.

In this study, even though the H-TPF group contained a lower proportion of patients with stage IV NPC than the L-TPF group (37.7% vs. 63.9%, *P* < 0.001), a PFS or OS benefit was not observed for H-TPF compared to L-TPF. Several factors may contribute to this result. More patients in the H-TPF group experienced treatment delays compared to the L-TPF group (33.3% vs. 19.4%, *P* = 0.034), which may allow tumor cell proliferation and offset any potential survival benefits of high-dose TPF. Previous studies conducted by Lee et al. [18] and Loong et al. [19] demonstrated the total dose of cisplatin administered during CCRT has a substantial effect on locoregional control and OS. In this study, fewer patients in the H-TPF group completed two cycles of concurrent cisplatin compared to the L-TPF group (81.2% vs. 100%, *P* < 0.001). Indeed, 26 patients in the H-TPF group did not receive their second cycle of concurrent chemotherapy due to hematologic adverse events (22 patients), non-hematologic adverse events (three patients) and patient refusal (one patient).

The doses received by the H-TPF group in this study are similar to the regimen used in two prospective phase 2 clinical trials registered online (National Clinical Trial [NCT] 00816855 for stage III NPC and NCT 00816816 for stage IVA-IVB NPC; 75 mg/m^2^ docetaxel on day 1, 75 mg/m^2^ cisplatin on day 1, and 2400 mg/m^2^ vs. 2500 mg/m^2^ continuous 24 h fluorouracil infusion for 4–5 days) [17]. Although the dose received by the L-TPF group in our study was nearly 20% lower than the conventional regimen used in the TAX323 study (60 mg/m^2^ vs. 75 mg/m^2^ docetaxel on day 1, 65 mg/m^2^ vs. 75 mg/m^2^ cisplatin on day 1, 550 mg/m^2^ vs. 750 mg/m^2^ fluorouracil per day on days 1–5) [20], the neoadjuvant chemotherapy regimen used in the L-TPF group was based on two phase 1–2 studies conducted at Sun Yat-sen University Cancer Center [21, 22]. Zhang et al. [21] found the maximum-tolerated dose (MTD) for 5-fluorouracil in locoregionally advanced NPC was 550 mg/m^2^ per day on days 1–5 when combined with 60 mg/m^2^ docetaxel and 60 mg/m^2^ cisplatin on day 1. Zhang Qun and colleagues [22] found the MTD of cisplatin in locoregionally advanced was 65 mg/m^2^ per day on day 1 NPC when combined with 60 mg/m^2^ docetaxel on day 1 and 550 mg/m^2^ 5-fluorouracil per day on days 1–5. The TAX323 and TAX324 trials indicated H-TPF neoadjuvant chemotherapy (75 mg/m^2^ docetaxel on day 1, 75–100 mg/m^2^ cisplatin on day 1, 3750–4000 mg/m^2^ continuous fluorouracil 24 h infusion for 4–5 days) induces a high rate of Grade 3 or 4 neutropenia (range, 56–76.9%) in patients with unresectable head and neck cancer [20, 23]. Even with G-CSF support and a lower dose of fluorouracil than the studies described above, Grade 3 or 4 neutropenia occurred in 55.2% of patients in the study conducted by Lin Kong et al. [17]. In this study, although Grade 3 or 4 neutropenia was similar in the H-TPF and L-TPF groups (63.8% vs. 73.6%, *P* = 0.149), more patients in the H-TPF group suffered Grade 4 neutropenia (43.4% vs. 6.9%, *P* < 0.001). Although all patients in both groups completed three cycles of neoadjuvant TPF, there were significantly more dose modifications and treatment delays in the H-TPF group, due to increased rates of Grade 4 neutropenia. There were no major differences in non-hematologic adverse events between the H-TPF and L-TPF groups during neoadjuvant chemotherapy. During chemoradiotherapy, more patients in the H-TPF group suffered grade 3 or 4 anemia, thrombocytopenia and neutropenia (*P* < 0.001, *P* < 0.001, *P* = 0.048; Table 4). Only 81.2% of patients in the H-TPF group completed two cycles of concurrent cisplatin, due to myelotoxicity and patient refusal. With the exceptions that esophagitis, dysphagia or odynophagia, and dry mouth were reported more often in the L-TPF group (which contained a higher proportion of patients with stage IV NPC), there were no major differences in non-hematologic adverse events between groups during chemoradiotherapy. Moreover, there were higher frequencies of grade 1 or 2 liver dysfunction and kidney dysfunction during chemoradiotherapy in the H-TPF group than L-TPF group, mainly due to the higher total dose of cisplatin in the H-TPF regimen.

This study has several limitations. Firstly, this was a retrospective study of a relatively small number of patients. Secondly, when we started this research, there was no consensus on the optimal IMRT dose fractionation in NPC. Thus, doctors at Zhejiang Cancer Hospital used a daily fraction of 2.1–2.3 Gy while doctors at Sun Yat-Sen University Cancer Center used a daily fraction of 2.1–2.12 Gy to the PTV of GTVnx and GTVnd. The optimal dose schedule for IMRT in NPC still needs further evaluation. Third, the H-TPF group contained a lower proportion of patients with stage IV NPC than the L-TPF group (37.7% vs. 63.9%, *P* < 0.001). Considering these limitations, the conclusions of this research need to be further validated.

In summary, this study suggests L-TPF neoadjuvant chemotherapy has substantially better tolerance and compliance rates than H-TPF neoadjuvant chemotherapy. Moreover, the treatment efficacy of L-TPF neoadjuvant chemotherapy and H-TPF neoadjuvant chemotherapy were similar. We recommend L-TPF neoadjuvant chemotherapy followed by CCRT is optimal for patients with locoregionally advanced NPC; however, long-term follow-up is required to evaluate the eventual treatment efficacy and side-effects of L-TPF neoadjuvant chemotherapy .

### Novelty and impact

We compared the efficacy and side-effects of neoadjuvant chemotherapy based on high-dose docetaxel, cisplatin and fluorouracil (H-TPF) versus low-dose TPF (L-TPF) in patients with locoregionally advanced nasopharyngeal carcinoma who receive subsequent chemoradiotherapy. L-TPF neoadjuvant chemotherapy had substantially better tolerance and compliance rates and a similar treatment efficacy compared to H-TPF neoadjuvant chemotherapy. We recommend L-TPF neoadjuvant chemotherapy followed by CCRT for patients with locoregionally advanced NPC.

## Abbreviations

(TPF): Docetaxel, cisplatin and fluorouracil
(PFS): progression-free survival
(OS): overall survival
(NPC): nasopharyngeal cancer
(CCRT): concurrent chemoradiotherapy
(NACT): neoadjuvant chemotherapy
(AJCC): American Joint Committee on Cancer
(IMRT): intensity modulated radiation therapy
(GTV): gross tumor volume
(CTV): clinical target volume
(3D-CRT): 3-dimensional conformal radiation therapy
(SCCHN): squamous cell carcinoma of the head and neck
(MTD): maximum-tolerated dose
(DMFS): distant metastasis free survival
